# 3D printed models in pregnancy and its utility in improving psychological constructs: a case series

**DOI:** 10.1186/s41205-022-00144-w

**Published:** 2022-06-09

**Authors:** John Joseph Coté, Brayden Patric Coté, Amy S. Badura-Brack

**Affiliations:** 1 Department of Obstetrics and Gynecology, CommonSpirit Health, Creighton University School of Medicine, 16909 Lakeside Hills Court, Suite 401, Omaha, NE 68130 USA; 2 Department of Psychological Science, Creighton University, 2500 California Plaza, Omaha, NE 68178 USA

**Keywords:** 3D printing, Maternal-fetal attachment, Paternal-fetal attachment, Anxiety, Depression, Twins, Facial cleft

## Abstract

**Background:**

3D printing is being utilized in almost every aspect of medicine. 3D printing has especially been used in conjunction with 3D ultrasonography to assist in antenatal assessment and presurgical planning with fetal malformations. As printing capabilities improve and applications are explored there may be more advantages for all parents to visualize and touch 3D printed models of their fetus.

**Case presentation:**

We present three cases involving 3D printed models and four different but interrelated psychological constructs- antenatal depression, antenatal anxiety, maternal-fetal attachment, and paternal-fetal attachment. Each case shows for the first time possible beneficial effects within these prevalent and significant problems.

**Conclusions:**

The degree to which the anxiety, depression, and attachment scores improved after the presentation of the 3D printed models is encouraging. Randomized controlled trials utilizing 3D printed models to improve psychological constructs should be supported considering the findings within these four cases.

## Introduction

Maternal-fetal attachment (MFA), paternal-fetal attachment (PFA), perinatal anxiety and depression are psychological constructs facing pregnant couples today. While the prevalence of maternal antenatal depression (20.7% 95% CI 19.4–21.9 [1]) and maternal anxiety (24.6% 95% CI 21.2–28.0 [2]) is well documented, MFA and PFA have been studied less frequently. The list of negative impacts on pregnant women, their fetuses and infants related to antenatal depression, anxiety and low MFA is staggering. These psychological factors are associated with placental abruption, gestational diabetes, pre-eclampsia/hypertension, preterm birth, intra-uterine fetal demise, and maternal death [3–13]. Pharmacologic therapies have been utilized to treat depression and anxiety successfully but their safety in pregnancy has been debated [14] and treatment with pharmacologic or non-pharmacologic interventions have not been proven to reduce the health risks commonly associated with antenatal depression or anxiety beyond psychological symptoms [15]. MFA and PFA have received even less clinical attention and research aimed at designing interventions to improve attachment scores in pregnancy [16, 17]. Recently a study showed that offering a third-trimester routine ultrasound does not have a psychological benefit for all pregnant women, yet it might improve MFA for women with higher levels of depressive symptoms [18]. A review also found that an MFA increase using various interventions can reduce induced anxiety and depression [19]. Studies have shown that fetal ultrasounds can improve MFA and while individual studies have not shown the utility of 3D ultrasonography over 2D ultrasonography, the trend favors the more accurate representation of the fetus [20–22].

3D printing technology has been reviewed for its utility in gynecology in a variety of instances: fibroids, endometriosis, pelvic floor disorders, gynecological oncology, patient education for surgical procedures, student education, and simulation-based training [23]. In obstetrics 3D printing technology has been used in cases of placenta accreta, fetal congenital anomalies for pre-surgical planning, and interactions with visually impaired pregnant patients [24–27]. Although we are unaware of any studies examining the impact of 3D printed technology on antenatal depression or anxiety, a few studies have shown significant improvement in MFA with 3D printed fetal models [27–29].

This report describes a series of three cases examining the use of 3D printed models’ utility in improving MFA, PFA, antenatal depression and anxiety.

## Cases

### Case one

**Fig. 1:**
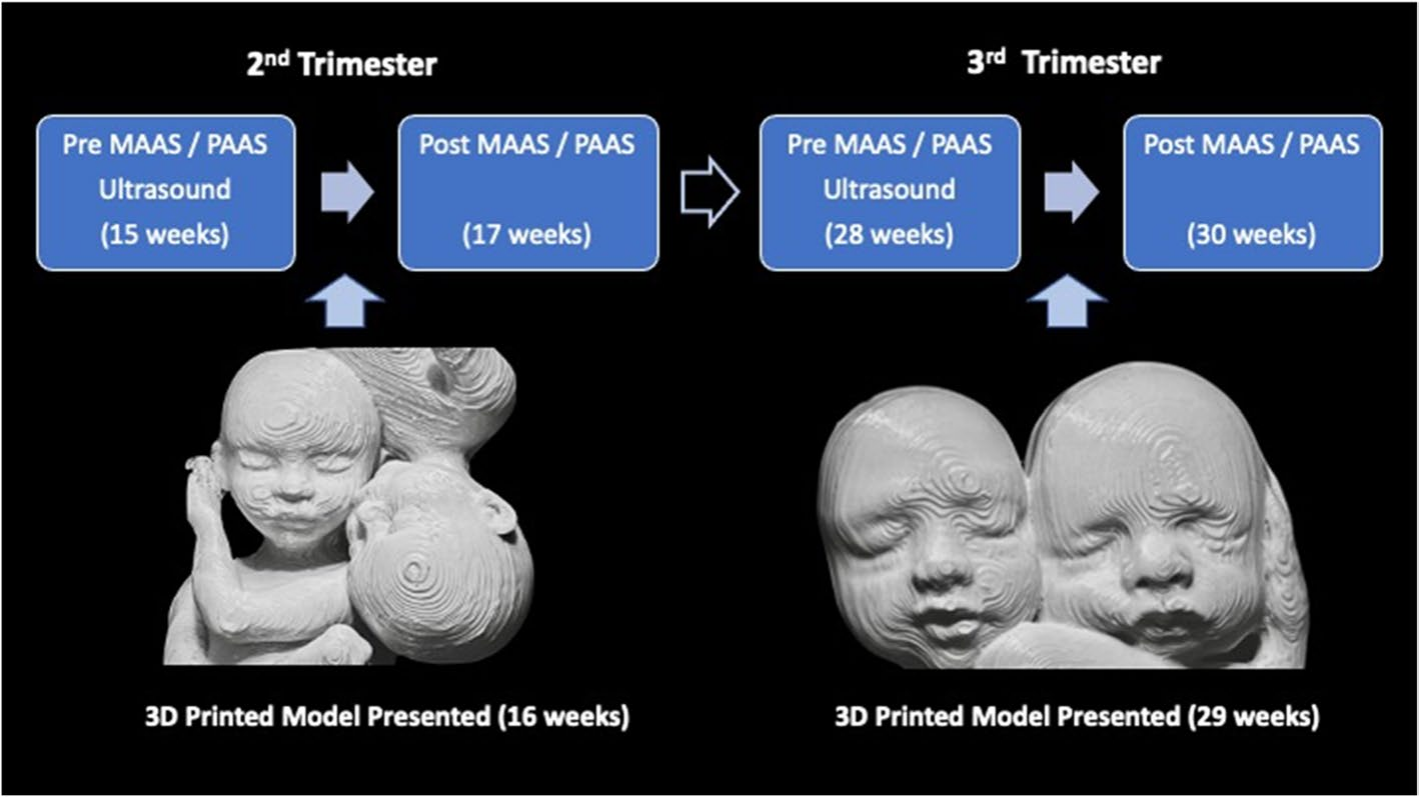
Timing of questionnaires and interventions throughout the pregnancy

A 35-year-old G3P1011 presented to the clinic for prenatal care with her 35-year-old husband who had fathered all her pregnancies and no others. An ultrasound performed at her initial visit showed a viable diamniotic/dichorionic twin pregnancy. Genetic amniocentesis and NIPT were declined. The patient and her husband were both healthy with no abnormal past medical or past surgical history. The patient had only been taking her prenatal vitamin and was started on a baby aspirin 81 mg. The patient and her husband provided consent for the case report, in accordance with Creighton Institutional Review Board (IRB) standards. The patients were told that she would need to answer the maternal antenatal attachment scale (MAAS) questionnaire and he would need to answer the paternal antenatal attachment scale (PAAS) [30, 31] at four different timepoints. The MAAS includes 19 statements and the PAAS includes 16 statements to which participants respond on a five-point Likert scale (1 = low and 5 = high). Therefore, global MAAS scores range from 19 to 95 while global PAAS scores range from 16 to 80. Both scales are reliable and valid, and the scales measure parents’ attitudes, feelings and behaviors toward the fetus [32]. There are items in both questionnaires that map to two subscale scores that measure the “quality of attachment” and “time spent in attachment”. Additionally, both parents would ‘journal their thoughts, feelings or emotions about the pregnancy and/or twins’. After consent was obtained the mother and father answered the MAAS and PAAS questionnaires independently and then underwent ultrasonography of the twins. The GE Voluson™ E10 exported the Digital Imaging and Communications in Medicine (DICOM) information and this was transferred to 3D slicer [33]. 3D Slicer segmentation of the volumetric data was performed, and the stereolithography (STL) file was loaded to the Cura Lulzbot® program. The Lulzbot TAZ Workhorse® [Material Extrusion] was used to print a model of both fetal faces together using polylactic acid (PLA) (Fig. 1A). One week after the initial study ultrasound examination the mother and father received a 3D-printed model of the twins produced from the ultrasonographic data. Both parents then answered the MAAS and PAAS questionnaires at 17 weeks (2 weeks after the initial ultrasound examination). At 28 weeks both parents independently answered the MAAS and PAAS questionnaires for the third time. They underwent a second study ultrasonography of the twins. The second 3D-printed model of the twins was presented to the mother and father a week later and the fourth MAAS and PAAS questionnaires were done at 30 weeks (2 weeks after the second study ultrasonography) (Fig. 1).

When the study concluded, both journals were transcribed separately into the IBM Watson™ Knowledge Studio on IBM Cloud® to perform sentiment and emotional analysis [34]. Watson Natural Language Understanding is a cloud native product that uses deep learning to extract metadata from text such as entities, keywords, categories, sentiment, emotion, relations, and syntax [35, 36]. Watson uses Unstructured Information Management Architecture which is a collection of analysis algorithms stacked to analyze a document [36]. Initially, each parent’s entire journal was analyzed. Then each parent’s journal was separated into the days of entry and each entry was analyzed individually.

Sentiment analysis is the computational study of opinions, sentiments and emotions expressed in text [37]. Watson uses a hybrid approach which incorporates linguistic and statistical analysis techniques [38]. Scores range from − 1 to 1, with − 1 being negative, 0 being neutral, and 1 being positive.

Emotional analysis recognises the emotion in a text. The Watson emotion score is created independently for five emotions: anger, fear, disgust, sadness, and joy. Scores range from 0 to 1 with higher scores indicating more of that emotion. Watson’s emotional categories are benchmarked against standard emotion data sets such as the International Survey on Emotion Antecedents (ISEAR) and Sematic Evaluation (SemEval) [39] and show excellent accuracy [40].

The MAAS scores were 85, 87, 90, 90 and the PAAS scores were 52, 55, 59, 65. Analyzing the journals revealed positive content for both parents. The maternal sentiment score was 0.63 and the paternal sentiment score was 0.53 on a scale of − 1.00 (negative) to + 1.00 (positive). Emotional analysis scores range from 0 to 1 to indicate the presence of the emotion. Emotional analysis of the entire maternal journal resulted in emotional scores of 0.64 ‘joy’, 0.60 ‘fear’, 0.56 ‘sadness’, 0.07 ‘anger’, and 0.05 ‘disgust’. Emotional analysis of the paternal journal revealed similarly high levels of 0.67 ‘joy’ and 0.57 ‘fear’, lower levels of 0.16 ‘sadness’, and similar negligible levels of 0.05 ‘anger’ and 0.03 ‘disgust’. Sample maternal diary entries mentioning the 3D printed models are listed below.

Today we got our 3-D print of the twins! Wow! This was so neat! We showed it to our family and sent pictures to some friends and everyone was in awe. I set the shadow box on the dresser in our bedroom so both [my husband] and I get to see it every day. This made me so happy and again, just helps to make it that much more real that we are actually having TWINS!

The sentiment appeared very positive at 0.85. Emotional analysis 0.61 ‘joy’, 0.07 ‘fear’, 0.11 ‘sadness’, 0.02 ‘disgust’ and 0.04 ‘anger’ paralleled the high sentiment.

Today was such a special day. [My toddler] has been taking notice of the 3-D shadow box of the twins on my dresser. For the past several days, he points to it and says “bebies”. . . today, [he] kept pointing to the box and wanted to hold it. We let him look at it and explore a bit. [My toddler] again pointed to the glass and said “bebies”, kisses. It was the sweetest thing I’ve ever seen.

The sentiment again was positive at 0.44. Emotional analysis was 0.56 ‘joy’, 0.07 ‘fear’, 0.54 ‘sadness’, 0.06 ‘disgust’, and 0.08 ‘anger’.

### Case two

**Fig. 2:**
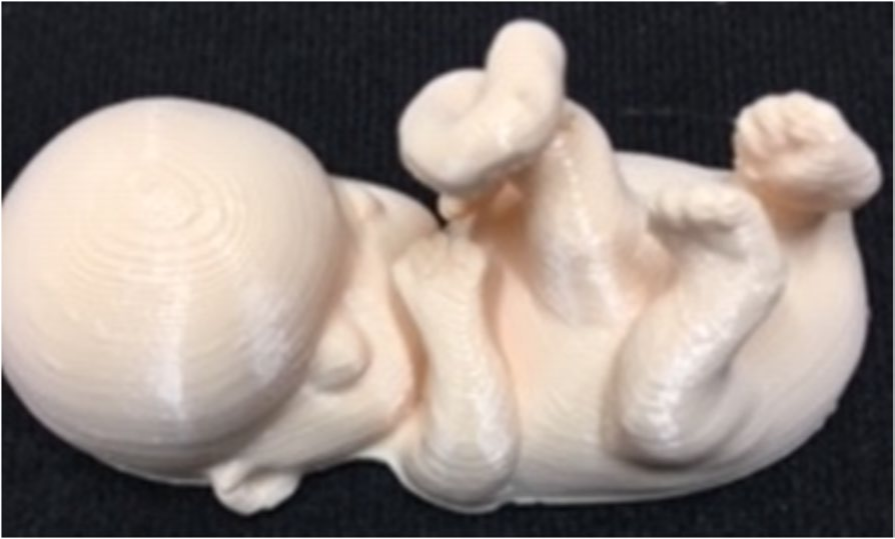
Eight-week-old fetus

A 21-year-old G3P0110 presented to clinic for prenatal care. An ultrasound performed at the initial visit showed a viable fetus at 8 weeks and 4 days. A 3D image was obtained at her early pregnancy ultrasound. Her history was significant for a 24-week delivery, due to severe pre-eclampsia, by cesarean section. The baby passed away on day of life 41 due to prematurity. She also had a previous early miscarriage at 9 weeks and 4 days. She had been taking Xanax® prior to the pregnancy for anxiety but had stopped after she had discovered she was pregnant. The patient provided consent for the case report, in accordance with Creighton IRB standards, at 12 weeks and 4 days. She was told she would need to answer a generalized anxiety disorder questionnaire (GAD-7) [41] and patient health questionnaire (PHQ-9) [42], at two time points. After consenting to the study and answering the questionnaires a 3D printed model of her 8-week-old fetus was presented to the patient (Fig. 2).

Taking the DICOM data from the 8-week ultrasound (GE Voluson™ E8) and converting it to STL created the image. The segmented image was printed on an Ultramaker 2 +® [Material Extrusion] using PLA [43]. One week after the presentation of 3D printed model, the patient answered the GAD-7 and PHQ-9 again.

The GAD-7 initial score was 13, moderately severe anxiety disorder. The answer to question number 8, “If you checked off any problems, how difficult have these problems made it for you to do your work, take care of things at home, or get along with other people” was “very difficult”. The PHQ-9 initial score was 19, moderately severe depression. Both anxiety and depression scores improved after the presentation of the 3D printed model. The GAD-7 score decreased to 6, mild anxiety disorder. The answer to the question number 8 changed to “somewhat difficult”. The PHQ-9 decreased to 7, mild depression.

### Case three

**Fig. 3:**
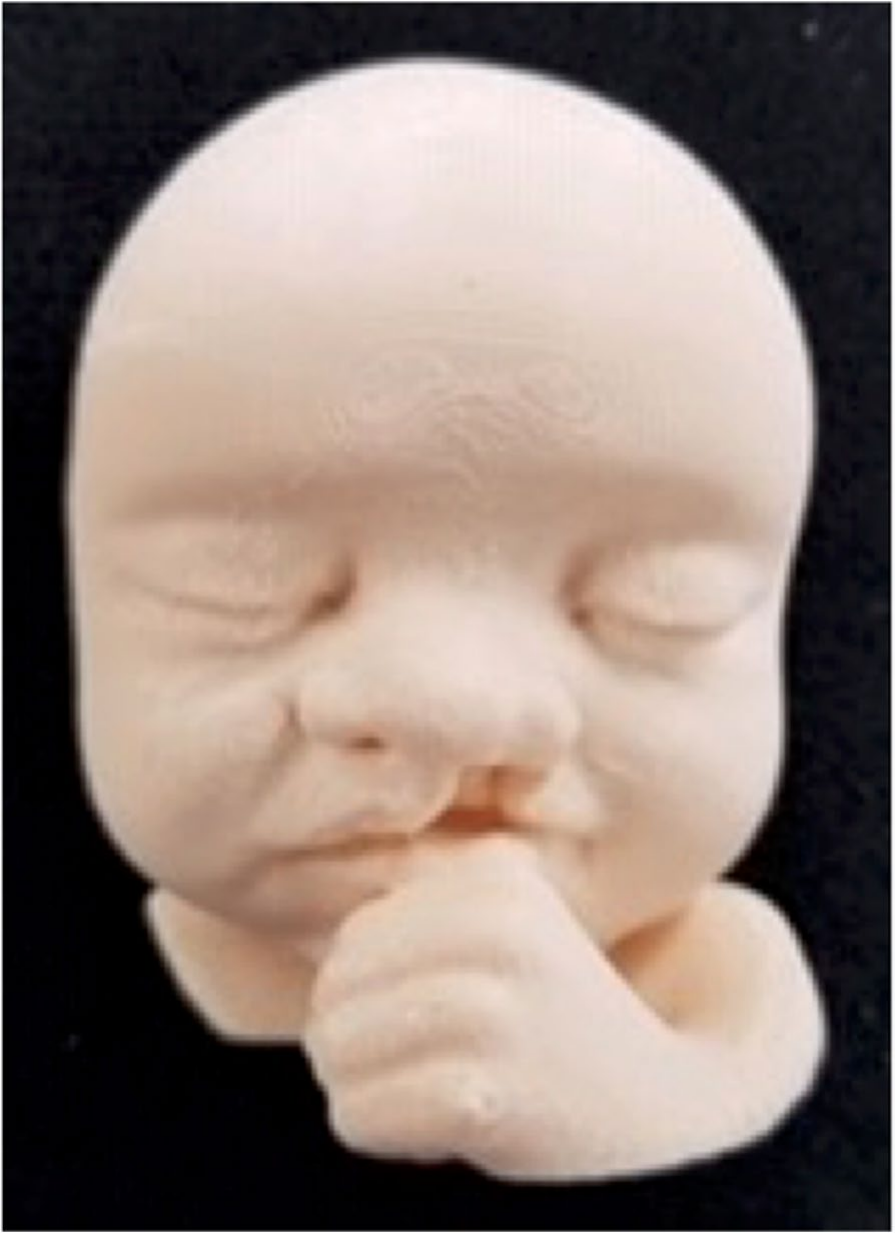
Fetus with facial cleft at 30-weeks

A 36-year-old G1P0 presented to clinic for prenatal care. An ultrasound performed at the 20-week fetal anatomic survey showed a fetus that had a suspected unilateral cleft lip and palate. Genetic amniocentesis and Non-Invasive Prenatal Testing (NIPT) were declined. No other anomalies were detected on follow-up ultrasounds. Patient had been taking Zoloft® during the pregnancy for depression. The patient provided consent for the case report, in accordance with Creighton IRB standards, at 30 weeks. She was told she would need to answer a PHQ-9 questionnaire and a MAAS questionnaire initially, and then again in 2 weeks. After consenting her, she completed the PHQ-9 and MAAS questionnaires. An ultrasonographer, who had neutral interactions, performed a 20-minute 3D/4D ultrasound (GE Voluson™ E10) and attempted to specifically target the fetus’ face. The patient was able to watch the screening in real-time on a large screen television. After the patient left the clinic, the DICOM data from the 3D ultrasound was extracted, segmented, cleaned, and converted to an STL file. The image was printed by an Ultimaker 2 +® [Material Extrusion] using PLA. One week after the initial ultrasound, the patient was asked to return to the clinic to receive a small shadow box containing the 3D printed model of her baby’s face produced from the ultrasound data (Fig. 3).

Finally, 2 weeks after the study ultrasound the patient completed the PHQ-9 and MAAS questionnaires again by phone.

The PHQ-9 initial score was 7, mild depression. The MAAS initial score was 65, with the time in attachment at 23 and the quality of attachment at 39. One week after the presentation of the 3D printed model the PHQ-9 score decreased to 4, minimal depression and the MAAS score increased to 77, with the time in attachment increasing to 27 and the quality of attachment increasing to 45.

## Discussion and conclusions

There are established links between a loss in a previous pregnancy and the current pregnancy with regards to anxiety, depression, and MFA [44, 45]. There is also evidence that 3D printed models may improve MFA, but this is the first time 3D printed models have been shown to potentially improve anxiety, depression and PFA within a pregnancy. Looking at depression, anxiety, and attachment through the lens of the 3D print as a type of non-pharmacologic treatment opens a vast array of applications for this technology. While researchers will look to transformative applications such as organ fabrication, customized prosthetics, and drug dosage and delivery applications for 3D printing [46] these are examples of current real time applications for ultrasounds and 3D printing that every obstetrician can begin to explore. A robust expanded cohort should be examined to determine if 3D printing of early pregnancies would help improve outcomes for women in pregnancies following a fetal or neonatal loss, and if 3D printed models can increase MFA and/or PFA in pregnancies, which include fetal facial clefts and twins.

## Data Availability

All data generated or analyzed during the current study are available from the corresponding author upon reasonable request.

## Abbreviations

MFA: Maternal-fetal attachment
PFA: Paternal-fetal attachment
MAAS: Maternal antenatal attachment scale
PAAS: Paternal antenatal attachment scale
GAD: Generalized anxiety disorder
PHQ: Patient health questionnaire
NIPT: Non-invasive prenatal testing
PLA: Polylactic acid
DICOM: Digital imaging and communications in medicine
IRB: Institutional review board
CI: Confidence interval
3D: Three dimensional
4D: Four dimensional
GE: General electric
STL: Stereolithography
IBM: International business machines
